# Risk Factors Associated with the Consumption of Sugar-Sweetened Beverages among Czech Adults: The Kardiovize Study

**DOI:** 10.3390/nu14245297

**Published:** 2022-12-13

**Authors:** Monika Kunzova, Geraldo A. Maranhao Neto, María M. Infante-Garcia, Ramfis Nieto-Martinez, Juan P. González-Rivas

**Affiliations:** 1 International Clinical Research Centre (ICRC), St Anne’s University Hospital Brno (FNUSA), 602 00 Brno, Czech Republic; 2 Department of Public Health, Faculty of Medicine, Masaryk University, 625 00 Brno, Czech Republic; 3 Foundation for Clinic, Public Health and Epidemiology Research of Venezuela (FISPEVEN INC), Barquisimeto 3001, Venezuela; 4 LifeDoc Health, Memphis, TN 38119, USA; 5 Departments of Global Health and Population and Epidemiology, Harvard TH Chan School of Public Health, Harvard University, Boston, MA 02115, USA

**Keywords:** sugar-sweetened beverages, soft drinks, Czechia, epidemiology, cardiovascular disease

## Abstract

High consumption of sugar-sweetened beverages (SSBs) is associated with a higher risk of cardiovascular disease (CVD). The last report on the prevalence of SSBs consumption in Czechia was 17 years ago, an updated analysis will enable the design of appropriate public health policies. This study aimed to determine the prevalence of SSBs consumption in a Czech city during 2020 and 2022, and its association with cardiometabolic biomarkers, behavioral risk factors, and socioeconomic determinants. A total of 730 participants (33 to 73 years) were assessed from a random population-based survey. SSBs consumption was evaluated using two methods: by calorie amount, with a 24 h dietary recall, and by frequency, with a food frequency questionnaire. By calorie amount, the prevalence of SSBs consumption was none: 52.5%, low: 30.0%, and moderate–high: 17.5%; by frequency was never: 16.0%, occasionally: 64.1%, and daily: 19.9%. SSBs intake was higher in men (*p* < 0.001) and younger participants (*p* = 0.001). Men consuming daily had higher waist circumference and visceral fat area compared to both occasional and never consumers. Higher SSBs consumption was associated with low household income, middle education level, and high total energy intake. In total, 20% drank SSBs daily and 17.5% of participants consumed moderate–high calorie amounts of SSBs. These results represent an increase in the prevalence of SSBs consumption in the last two decades. Public health policies should target men of younger age and people with low education and income.

## 1. Introduction

The consumption of sugar-sweetened beverages (SSBs) is associated with a higher body mass index (BMI) [1,2,3] and higher risk of cardiometabolic disease—including type 2 diabetes (T2D) and cardiovascular disease (CVD)—and oncologic disease [4]. Worldwide, in 2010, 184,000 deaths were attributed to the consumption of SSBs: 133,000 from T2D, 45,000 from CVD, and 6450 from cancer [5]. SSBs increase abnormal adiposity through varied mechanisms, increasing energy intake, calibrating the preferences to a higher level of sweetness [6], providing incomplete compensation for the extra energy consumed in SSB via reducing solid energy intake [7], and decreasing satiety [2,4]. In addition, higher consumption of SSBs has been linked with a deleterious genetic effect on adiposity—daily consumers were more genetically susceptible to effects on the risk of obesity and BMI than those who consumed SSBs less than once per month [8]. Due to their high glycemic index, which facilitates abnormal adiposity and a rapid increase in blood glucose level after consumption, SSBs can impair the normal glucose metabolism T2D [9]. SSBs consumption is also associated with higher blood pressure and increased uric acid levels.

The pattern of SSBs consumption differs by country [10], sex, age, and socioeconomic determinants [5]. From 2009–2014, global sales of SSBs remained unchanged [10]. In Europe, sales of SSBs decreased in western countries and increased in eastern countries [10]. In 2019, the European Health Interview Survey (EHIS) reported that the leading countries with the highest daily consumption were Belgium (20.4% of the population drink SSBs, defined as “heavy-sugared, often carbonated lemonades”, once or more per day), Malta (12.4%), Germany (12.1%), Hungary (12.0%), Poland (12.0%), Bulgaria (11.6%), and Czechia (11.4%) [11,12]. According to the HAPIEE (Health, Alcohol and Psychosocial Factors in Eastern Europe) study, conducted from 2002 to 2005 in Russia, Poland, and Czechia, subjects who drank SSBs daily (defined as non-alcoholic carbonated [fizzy] drinks, such as coke, fizzy orange, or lemonade) had a higher BMI [3]. In this study, regular SSBs intake was more common in men, younger subjects, lower education level, lower physical activity, regular alcohol drinkers, and smokers [3].

Even though many countries implement and consider specific steps to reduce SSBs consumption, most of the main stakeholders and general local authorities have insufficient awareness [13]. Providing timely information on the prevalence and impact of SSBs consumption facilitates the implementation of appropriate public health policies. This study aims to analyze the prevalence of SSBs consumption and the association with cardiometabolic, behavioral risk factors, and socioeconomic determinants, in a randomly selected sample of middle-aged adults from Czechia, evaluated during 2020–2022. Following the definition of the World Health Organization, which considers SSBs as any liquid containing free sugars, in the present study, the SSBs and fruit juices that also provide free sugars were analyzed together [14].

## 2. Materials and Methods

### 2.1. Study Design and Population

The Kardiovize study design, sampling, and implementation were described previously [15]. In brief, the Kardiovize Brno 2030 is an ongoing multidisciplinary epidemiological project designed as a cohort population-based study with a random sample of 1% of the adult population of Brno aged between 25 and 64 years old [15]. Brno is the second largest city in the Czech Republic, with 380,654 residents in 2019 [16]. The eligibility criteria included permanent residence in Brno and registration (required by the law) with any of the five state-run health insurance companies operating in Czechia. The recruitment and core baseline examinations were completed in 2014 (n = 2160), and the first follow-up was implemented from 2020 to 2022. For the follow-up evaluation, three invitations were sent (two by mail and one by phone), and 755 participants attended the follow-up evaluation (response rate 34.9%). Twenty-five participants were excluded from the present analysis due to either missing information or outlying values.

### 2.2. Data Collection

The health assessment, face-to-face health interview, and comprehensive questionnaire were performed by trained nurses, dietitians, and physicians at the International Clinical Research Center of the St Anne’s University Hospital in Brno. The data was entered into the web-based research electronic data capture (REDCap; Vanderbilt University, Nashville, TN, USA) database [17]. The questionnaire included demographics (e.g., age, sex, and education), socioeconomic status (e.g., household income), cardiovascular risk behaviors (e.g., smoking habit, alcohol consumption, and physical activity), family and personal history, medications, and hospitalizations. In a food frequency questionnaire, participants were asked, “How often, on average, in the last 3 months have you consumed specific foods and drinks?”. In a 24 h recall questionnaire administered by dietitians, participants were asked to report all individual food components consumed the day before (along with specific ingredients). When possible, the exact weights reported by participants were recorded, or dietitians recorded standardized portions or weights of foods. Dietary data were calculated in the NutriPro Expert software (version 9.3.1.0; Fitsport-komplex Ltd., Ivančice, Czechia) [18]. Laboratory analyses were performed on 12 h fasting whole blood samples using a Modular SWA P800 analyzer (Roche, Basel, Switzerland). The total cholesterol, triglycerides, and glucose were analyzed by the enzymatic colorimetric method (Roche Diagnostics GmbH). High-density lipoprotein cholesterol (HDL-c) was analyzed with the homogeneous method for direct measurement without precipitation (Sekisui Medical). Low-density lipoprotein cholesterol (LDL-c) level was calculated according to the Friedewald equation when triglyceride levels were below 4.5 mmol/L; if it was higher, LDL-c was analyzed using the homogeneous method for direct measurement (Sekisui Medical). Blood pressure was measured with the patient alone using an automated office measurement device (BpTRU, model BPM 200; Bp TRU Medical Devices Ltd., Canada). Height and weight were measured using a medical digital scale with a meter (SECA 799; SECA^®^, GmbH and Co. KG, Hamburg, Germany) and waist, hip, and neck circumferences were measured with a manual tape. Weight and body composition analyses were performed with a balance with bioelectrical impedance analysis (InBody 370; BIOSPACE Co., Ltd., Seoul, Republic of Korea).

### 2.3. Variables Definition

In this analysis, SSBs were considered as liquid beverages with added sugar, including water, sweetened coffee and tea, carbonated soft drinks, fruit drinks and nectars, sports drinks, and energy drinks. Two methods of nutritional assessment were used: a 24 h recall (assessed by calorie amount) and a Food Frequency Questionnaire (FFQ–assessment by frequency). Using both methods ameliorates their limitations. The 24 h recall assesses all food choices, and the querying of a trained dietitian is less demanding on the memory, but it only assesses the previous day’s consumption. The FFQ provides a comprehensive overview of current consumption over a long period, but limitations are the participant’s memory and estimation of beverage consumption with limited food items [19]. A 24 h recall was used to categorize participants with high, moderate, low, and no consumption of SSBs. Using the World Health Organization Guideline [20], categories were based on energy intake from added sugar from SSBs and participants were stratified into three categories: none (consume <1% of total energy intake as added sugar from SSBs), low (1% to <5%), and moderate–high (≥5%). To determine the frequency of consumption, an FFQ with all types of SSBs mentioned above was used. Participants were asked: “How often, on average in the last 3 months have you consumed specific foods and drinks?”. For all SSBs, one drink was equivalent to 200 mL. Participants were classified into three categories of frequency: never, occasional (<1 SSB per day), and daily (≥1 SSBs per day). Three categories were specified to assess the proportion of the potential effect and not just the difference between drinkers and non-drinkers [3]. Total energy intake was assessed from a 24 h recall. For this analysis, all participants were classified into three categories of their total energy intake in kcal per day (<2000; 2000–2500; >2500) [3]. Alcohol intake was obtained from a 24 h diet recall, processed in NutriPro software as grams of ethanol per day, and classified as: none (consume 0 g of ethanol per day), middle (>0–20 g/day), and high (>20 g/day) [3]. Hypertension was defined as systolic blood pressure ≥ 140 mmHg or diastolic blood pressure ≥ 90 mmHg or personal history of hypertension or use of antihypertensive medication [7]. Smoking status was categorized as never smokers, or those having smoked fewer than 100 cigarettes in a lifetime; past smokers, those having stopped smoking at least a year ago; and current smokers, those who smoke either daily or less than daily over the past year. Physical activity was assessed using the International Questionnaire of Physical Activity (IPAQ) long version [21]. Participants categorized as active participated in a vigorous-intensity activity on at least 3 days, achieving a minimum of at least 1500 MET-minutes/week, or 7 or more days of any combination of walking, moderate-intensity, or vigorous-intensity activities, achieving a minimum of at least 3000 MET-minutes/week. Participants categorized as minimally active participated in 3 or more days of vigorous activities of at least 20 min per day, 5 or more days of moderate-intensity activities or walking of at least 30 min per day, or 5 or more days of any combination of walking, moderate-intensity, or vigorous-intensity activities achieving a minimum of at least 600 MET-min/week [21]. Participants categorized as inactive did not participate in any of the activities above. Education was categorized as low (primary school even not completed and an apprenticeship with/without a school-leaving exam), medium (Secondary school with a school-leaving exam), and high (post-secondary specialized school, University, or similar) [22]. Household income was expressed in Euros per month and classified as low (<1200), middle (1200–1800), or high (>1800).

### 2.4. Ethics Approval

The study protocol complied with the Helsinki declaration and all participants signed an informed consent form. The Kardiovize study was approved by the ethics committee of St Anne’s University Hospital, Brno, Czech Republic (Ref. Number: 28V/2019).

### 2.5. Statistical Analysis

The SPSS software (SPSS, version 28.0.0.0, IBM Corp.) was used. Considering calorie amount classification, participants were stratified into four categories—none, low, moderate, and high. Because the number of respondents in the categories moderate and high was low, 12.45%, and 5.06%, respectively, these categories were merged into moderate–high. Continuous variables were presented as mean and standard deviation. The two-sample *t*-test was used to determine the difference between the means. Proportions were presented as percentages and 95% confidence interval (95% CI). The chi-square test was used to determine the difference between proportions. Differences between groups were assessed using the ANOVA for continuous parameters and Fisher’s exact or chi-square test for categorical parameters. Multinomial logistic regression analysis was used to determine behavioral and social factors related to the different SSBs consumption patterns, adjusting each variable by age and sex, and then fully adjusted by all covariates. The outcome variable was SSBs consumption by the amount and by frequency. The level of statistical significance was set at *p* < 0.05.

## 3. Results

### 3.1. Participants’ Characteristics

In total, 730 participants were included, with a mean age of 55.2 ± 10.8 years, and 52.6% were women. Men had higher weight, BMI, waist circumference, systolic blood pressure, diastolic blood pressure, fasting blood glucose, triglycerides, lower HDL-c, and a higher prevalence of hypertension, active physical activity level, higher alcohol intake, and higher total energy intake than women (Table 1). Women had a higher visceral fat area, total cholesterol levels, and a higher prevalence of current smoking than men. Men reported higher educational levels and higher household income than women (Table 1).

### 3.2. Prevalence of SSBs Consumption

#### 3.2.1. By Calorie Amount

**Figure 1:**
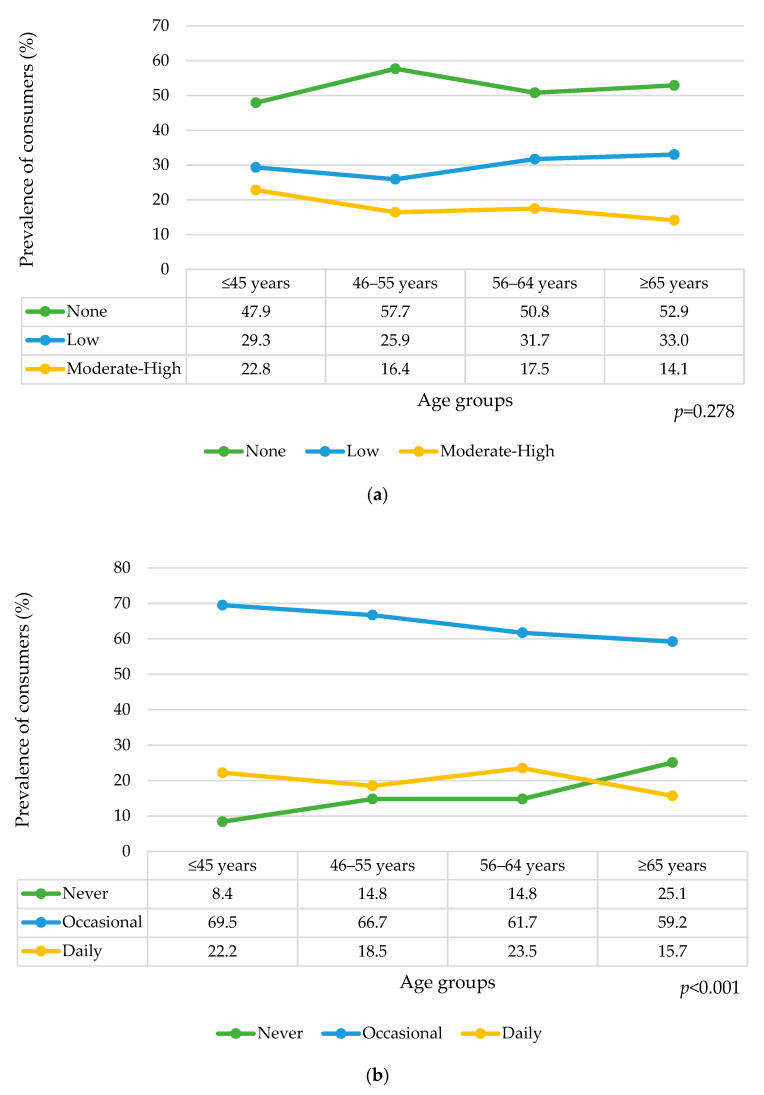
Prevalence of SSBs consumption in the sample stratified by age groups: (**a**) by calorie amount classification; (**b**) by frequency classification.

The prevalence of SSBs consumption was none: 52.5% (95% CI: 49.0–55.9); low: 30.0% (95% CI: 26.4–33.7); and moderate–high: 17.5% (95% CI: 14.8–20.3) (Table 2). “None-consumption” was higher in women than men: 57.3% (95% CI: 52.6–62.8) and 47.1% (95% CI: 42.1–52.3), respectively (*p* < 0.001), whereas moderate–high consumption was higher in men than women: 23.4% (95% CI: 19.0–28.0) and 12.2% (95% CI: 9.0–15.7), respectively, (*p* < 0.001; Table 2). The amount of SSBs consumed was similar among age groups (*p* = 0.278; Figure 1a).

#### 3.2.2. By Frequency

The prevalence of SSBs consumption was never: 16.0% (95% CI: 13.3–18.8); occasional: 64.1% (95% CI: 60.7–67.3); and daily: 19.9% (95% CI: 17.0–22.7) (Table 2). “Never consumption” was higher in women than men, 19.8% (95% CI: 15.7–23.9) and 11.8% (95% CI: 8.3–15.3), respectively (*p* < 0.001), whereas daily consumption was higher in men than women: 28.3% (95% CI: 23.8–33.1) and 12.2% (95% CI: 8.9–15.6), respectively (*p* < 0.001; Table 2). The frequency of SSBs consumption was different by age groups (*p* = 0.001). The prevalence of never consumption increased by age, from 8.4% (95% CI: 4.3–13.0) in those younger than 45 years old, to 25.1% (*p* < 0.001) in the oldest (Figure 1b). Occasional and daily consumption remained similar by age groups.

### 3.3. SSBs Consumption and Cardiometabolic Biomarkers

#### 3.3.1. By Calorie Amount

In both sexes, the value of cardiometabolic biomarkers, including waist circumference, visceral fat area, blood pressure, total cholesterol, HDL-cholesterol, LDL-cholesterol, triglycerides, and fasting blood glucose were similar among the three categories of the SSBs amount of consumption (Table 3).

#### 3.3.2. By Frequency

In men, daily consumers had a higher waist circumference (101.9 ± 11.9 cm) than occasional and never consumers (96.8 ± 11.2 cm and 96.6 ± 12.5 cm, respectively, *p* = 0.001), and a higher visceral fat area (104.7 ± 40.1 cm^2^) than occasional and never consumers (90.3 ± 40.4 cm^2^ and 92.9 ±48.6 cm^2^, respectively, *p* = 0.018). In women, the values of cardiometabolic biomarkers were similar among the diverse frequencies of consumption (Table 3).

### 3.4. SSBs Consumption and Behavioral Risks Factors

#### 3.4.1. By Calorie Amount

In men, the SSBs amount consumed was not associated with behavioral risk factors, including physical activity, smoking, alcohol intake, and total energy intake (Table 4). Women with no consumption had a higher prevalence of lower total energy intake than those with low and moderate–high consumption (*p* = 0.023; Table 5). After adjusting by age and sex, using multinomial logistic regression analysis with no consumption as a reference, low consumption was associated with a higher total energy intake (Table 6). Using a fully adjusted model with no consumption as a reference, low consumption was associated with a higher total energy intake; moderate–high consumption was associated with a high alcohol intake and higher total energy intake (Table 7).

#### 3.4.2. By Frequency

Men and women with lower total energy intake had a higher prevalence of never consumption than those with higher total energy intake (*p* < 0.05; Table 5). In women, daily consumers had a lower prevalence of alcohol intake than those with lower SSBs consumption (*p* = 0.039; Table 5). After adjusting by age and sex (Table 6) and adjusting by all covariates (Table 7) using multinomial logistic regression with never frequency as a reference, occasional consumption was associated with minimal physical activity, but the result was not consistent with daily consumption. Daily consumption was associated with higher total energy intake.

### 3.5. SSBs Consumption and Socioeconomic Determinants

#### 3.5.1. By Calorie Amount

In men, the SSBs amount consumed was not associated with socioeconomic determinants, including household income and education level (Table 4). Women with no consumption had a higher prevalence of high education levels than those with low and moderate–high consumption (*p* = 0.019; Table 5). After adjusting by age and sex (Table 6) and adjusting by all covariates (Table 7), using multinomial logistic regression with no consumption as a reference, moderate–high consumption was associated with low household income. Low and moderate–high consumption were associated with middle education levels.

#### 3.5.2. By Frequency

In men, lower household income was associated with never consumption (*p* = 0.026; Table 4). In women, the prevalence of consumption was similar among the social determinants (Table 5). After adjusting by age and sex (Table 6) and adjusting by all covariates (Table 7), using multinomial logistic regression with never frequency as a reference, daily consumption was associated with middle education level.

## 4. Discussion

This study aimed to analyze the prevalence of SSBs consumption and the association with cardiometabolic, behavioral risk factors, and socioeconomic determinants. In total, 17.5% of participants consumed moderate–high amounts of SSBs, and 19.9% drank SSBs every day. Evaluating the consumption of SBBs by calorie amount and frequency, men reported a higher amount and frequency of consumption than women. Despite the amount consumed being similar by age group, younger participants reported a higher frequency of consumption than older adults. The amount of consumption was not associated with the presence of cardiometabolic biomarkers, but men with a higher frequency of consumption had higher waist circumference and visceral fat adiposity than those with a lower frequency of consumption. There was no clear association between SBBs consumption and behavioral risk factors, in general, a lower consumption (in women) and lower frequency (in both sexes) were associated with a lower energy intake. The lower amount of consumption was associated with higher education levels in women, and a higher frequency was associated with lower household income in men.

Compared to the Czech results of the HAPIEE study, which reported the prevalence of consumption by frequency, in this study, never consumption was lower by 30.8% (46.8% vs. 16.0%, respectively), occasional consumption (<1 drink per day) was higher by 33.3% (30.8% vs. 64.1%, respectively), and daily consumption (≥1 drink per day) was lower by 2.5% (22.4% vs. 19.9%, respectively). Despite the inherent limitations comparing both studies, considering that in the HAPIEE study only non-alcoholic carbonated drinks (such as coke, fizzy orange, or lemonade) were included, these results represent an apparent increase in the frequency of consumption of SSBs in the Czech population in the last 17 years. Additionally, compared with the three countries included in the HAPIEE study (exclusive results from Czechia were not available), men reported higher frequency than women. Similarly, lower education levels, regular alcohol intake, and lower physical activity were associated with a higher frequency of consumption [3], which is consistent with the results of this study.

Men who consumed SSBs daily had higher waist circumference and visceral fat area compared to occasional drinkers and non-consumers. These findings are consistent with other epidemiological studies, where the consumption of SSBs has been associated with adverse effects on ectopic and visceral fat accumulation [23,24]. SSBs are sweetened with high-fructose corn syrup, which is 45–58% glucose and 42–55% fructose; or sucrose which is 50% glucose and 50% fructose [24]. Excess fructose intake could lead to atherogenic dyslipidemia, insulin resistance, and increased hepatic de novo lipogenesis. SSBs promote the production and secretion of very low-density lipoproteins leading to increased concentrations of postprandial triglycerides [25]. This mechanism stimulates the deposition of triglycerides in visceral adipose tissue mediated by lipoprotein lipase, which is regulated by insulin. It was proposed that excess fructose intake from SSBs results in hepatic fat accumulation, leading to hepatic and peripheral insulin resistance [23]. These mechanisms link visceral fat accumulation, SSBs consumption, and increased risk of prediabetes and T2D [6,26].

High intake of sugar from SSBs has been associated with adverse gut microbiota composition; however, the mechanism is not clear. A potential pathway is the malabsorption of fructose, one of the most poorly absorbed carbohydrates. Unabsorbed fructose in excessive loads passes to the large intestine where it becomes a fermentable substrate for the gut microbiota. As a result, the composition of the microbiota is modified according to the available substrate and could cause dysbiosis with loss of microbial diversity [27]. Additionally, a higher *Firmicutes: Bacteroides* ratio, which has been several times associated with overweight and obesity [28], was also positively associated with SSBs intake [29].

Effective public health interventions to reduce SSBs consumption include the following tools: traffic light labeling of products, in-store promotion of healthier beverages in supermarkets, government food benefit programs with restrictions on purchasing SSBs, multicomponent community campaigns focused on SSBs, or improved availability of low-calorie drinks in the home environment [30]. According to best buys by the World Health Organization, an efficient intervention to reduce sugar consumption is through effective taxation on sugar-sweetened beverages [31]. The global health policy recommendation of an adaptation of SSBs taxes, with a constructive collaboration between finance and health policymakers as a key factor, is operating in many European countries, such as Belgium, France, Hungary, Netherlands, and the UK. Both tax bases and rates vary between states, and the usual form of taxes is excises or levies [32]. As evidenced by UK data from 2015–2018, sales of taxable SSBs fell by 50% due to the implementation of the soft drink industry levy [33]. Model-based analysis performed in Germany implementing a 20% SSBs tax showed that this strategy could have a significant impact on overweight and obesity [34]. Public acceptability is higher when the income from taxes is used for health initiatives [35]. Those strategies could be an integral part of an effective action against the obesity pandemic. In Czechia, a special policy on unhealthy foods that sets out the requirements for food, including beverages, that can be offered for sale and sold in schools and educational establishments, started in 2016 [36]. Regulations covering the whole Czech population are not yet available.

One limitation of the present study that can be highlighted is the cross-sectional nature, which does not allow for the determination of causality. Therefore, reverse causality may play a role in some observed relationships. Second, measurements of SSB intake by FFQ and just a 24 h recall of a day’s intake may be inaccurate and lead to misclassifications [37]. Furthermore, the urban character of the study sample does not allow the generalization of the findings to the entire population. Responders could probably be healthier compared to the general population [38]. Comparing our data with other studies due to the different definitions of SSBs was difficult. Most studies define SSBs as beverages with sugars that are added during processing, such as carbonated and non-carbonated lemonades. These studies excluded beverages with naturally occurring sugars, such as 100% fruit juices. Although WHO guidelines specify that 100% fruit juices provide free sugars, these drinks are still generally perceived as healthier choices by consumers. Regarding differences between types of sugar, consumers should be aware that even 100% juices contain as much sugar (naturally present) and energy as carbonated beverages [39]. On the other hand, the extensive and rigorous measurement of risk factors and the participation of a trained multidisciplinary team increase the study’s validity. The main strength of the study is that it presents information on the current prevalence of SSB consumption in Czechia with associated cardiometabolic biomarkers, behavioral risk factors, and socioeconomic determinants using two validated measurements.

## 5. Conclusions

In total, 20% of participants drank SSBs daily, with higher consumption in men than in women. Daily consumption was associated with greater waist circumference and visceral fat area in men. Compared to previous Czech reports, these results represent that the prevalence of occasional drinking (<1 drink per day) has doubled in the past decade, and the daily drinking remains unchanged. However, 20% of daily consumption nearly doubles the prevalence reported by the European Health Interview Survey (EHIS–11.4%). Using an additional metric, 17.5% of participants consumed moderate-to-high calorie amounts of SSBs. These results demand a call to action to establish public health policies to reduce alcohol consumption, targeting mainly younger men and individuals with low education and income. The implementation of policies with evidence-based interventions could reduce the consumption of SSBs, and the promotion of healthier beverage reformulations would bring benefits to the population’s health.

## Figures and Tables

**Table 1:** Participants’ characteristics.

		Men	Women	Total	*p*-Value
Participants, n (%)		346 (47.4)	384 (52.6)	730 (100)	
Age (years)		54.5 (10.9)	55.9 (10.6)	55.2 (10.8)	0.079
Weight (kg)		87.7 (13.9)	72.1 (15.1)	79.5 (16.5)	<0.001
Height (cm)		180.4 (7.1)	167.1 (6.5)	173.4 (9.5)	<0.001
BMI (kg/m^2^)		26.9 (4.0)	25.8 (5.4)	26.3 (4.8)	0.001
Waist circumference (cm)		98.2 (11.7)	87.0 (13.5)	92.3 (13.9)	<0.001
Visceral fat area (cm^2^)		94.7 (41.8)	108.4 (51.8)	101.9 (47.8)	<0.001
Systolic Blood Pressure (mmHg)		120.1 (13.6)	118.0 (15.8)	119.0 (14.8)	0.047
Diastolic Blood Pressure (mmHg)		78.6 (8.7)	74.3 (8.9)	76.4 (9.1)	<0.001
Total cholesterol (mmol/L)		5.2 (0.9)	5.4 (0.9)	5.3 (0.9)	0.004
HDL-cholesterol (mmol/L)		1.3 (0.3)	1.7 (0.4)	1.5 (0.4)	<0.001
LDL-cholesterol (mmol/L)		3.3 (0.9)	3.3 (0.9)	3.3 (0.9)	0.630
Fasting Triglycerides (mmol/L)		1.4 (0.7)	1.1 (0.5)	1.2 (0.7)	<0.001
Fasting blood glucose (mmol/L)		5.5 (1.1)	5.2 (0.8)	5.3 (1.0)	<0.001
Hypertension		41.6 (36.7–46.8)	31.5 (27.3–36.2)	36.3 (32.9–39.4)	0.005
Physical activity level	Active	46.2 (40.9–51.7)	40.1 (35.1–44.9)	43.0 (39.5–46.6)	
	Minimally active	36.7 (31.4–41.9)	43.8 (38.9–48.8)	40.4 (37.0–44.1)	0.141
	Inactive	17.1 (13.1–21.1)	16.1 (12.6–19.9)	16.6 (14.1–19.3)	
Smoking habit	Never smoker	14.7 (11.3–18.6)	15.1 (11.6–18.6)	14.9 (12.3–17.5)	
	Past smoker	32.4 (27.4–37.3)	26.3 (22.1–30.7)	29.2 (25.8–32.6)	0.185
	Current smoker	52.9 (47.8–58.0)	58.6 (54.0–63.5)	55.9 (52.3–59.5)	
Alcohol intake	None	59.5 (54.5–65.0)	70.3 (65.5–74.9)	65.2 (61.6–68.8)	
	Middle	16.2 (12.4–19.9)	19.3 (15.4–23.2)	17.8 (15.1–20.8)	<0.001
	High	24.3 (19.9–28.9)	10.4 (7.6–13.6)	17.0 (14.1–19.7)	
Total energy intake (kcal/day)	<2000	42.8 (37.5–47.7)	72.4 (68.1–76.7)	58.4 (54.8–62.1)	
	2000–2500	28.0 (23.2–32.9)	20.6 (16.6–24.8)	24.1 (21.1–27.4)	<0.001
	>2500	29.2 (24.5–34.3)	7.0 (4.6–9.6)	17.5 (14.8–20.3)	
Education level	High	51.4 (46.5–56.4)	38.8 (34.0–43.6)	44.8 (41.8–48.1)	
	Medium	28.0 (23.2–32.9)	39.6 (34.5–44.5)	34.1 (30.7–37.8)	0.001
	Low	20.5 (16.1–25.0)	21.6 (17.8–25.7)	21.1 (18.4–24.1)	
Household income	High	37.3 (32.1–42.2)	18.0 (14.4–22.1)	27.1 (24.0–30.3)	
	Middle	48.3 (42.8–53.5)	49.2 (44.3–54.3)	48.8 (45.2–52.6)	<0.001
	Low	14.5 (10.8–18.2)	32.8 (28.1–37.6)	24.1 (21.1–27.1)	

Continuous variables are presented as mean and standard deviation. The two-sample *t*-test is used to determine different medians. Proportions are presented as percentages and a 95% Confidence Interval (95% CI). The chi-square test is used to determine different proportions.

**Table 2:** Prevalence of SSBs consumption in the sample in general and stratified by sex.

SSBs Consumption	Men	Women	Total	*p*-Value
By calorie amount				
None	47.1 (42.1–52.3)	57.3 (52.6–62.8)	52.5 (49.0–55.9)	<0.001
Low	29.5 (24.9–34.3)	30.5 (25.4–35.0)	30.0 (26.4–33.7)	
Moderate–High	23.4 (19.0–28.0)	12.2 (9.0–15.7)	17.5 (14.8–20.3)	
By frequency				
Never	11.8 (8.3–15.3)	19.8 (15.7–23.9)	16.0 (13.3–18.8)	
Occasional	59.8 (55.0–64.9)	68.0 (63.2–72.5)	64.1 (60.7–67.3)	<0.001
Daily	28.3 (23.8–33.1)	12.2 (8.9–15.6)	19.9 (17.0–22.7)	

Proportions are presented as percentages and a 95% Confidence Interval (95% CI). The chi-square test is used to determine different proportions. Abbreviation: SSBs—Sugar-sweetened beverages.

**Table 3:** Description of the values of cardiometabolic biomarkers by SSBs consumption patterns (by calorie amount and by frequency) stratified by sex.

By Calorie Amount						By Frequency			
	Sex	None	Low	Moderate–High	*p*-Value	Never	Occasional	Daily	*p*-Value
Waist circumference (cm)	M	98.2 (11.2)	96.9 (12.9)	99.9 (11.1)	0.222	96.6 (12.5)	96.8 (11.2)	101.9 (11.9)	0.001
	W	87.1 (14.1)	85.5 (11.7)	90.0 (14.4)	0.152	85.3 (13.8)	86.8 (12.6)	90.7 (17.0)	0.092
Visceral fat area (cm^2^)	M	95.6 (43.6)	90.3 (41.2)	98.4 (38.5)	0.397	92.9 (48.6)	90.3 (40.4)	104.7 (40.1)	0.018
	W	109.5 (53.3)	103.5 (49.1)	116.7 (50.8)	0.317	105.4 (56.2)	107.2 (48.6)	121.1 (60.6)	0.200
Systolic Blood Pressure (mmHg)	M	120.3 (13.3)	118.7 (14.3)	121.6 (13.4)	0.348	121.8 (14.8)	119.4 (13.2)	121.0 (14.0)	0.468
	W	118.8 (16.7)	115.9 (13.9)	118.9 (15.7)	0.238	117.9 (17.0)	117.8 (15.7)	118.8 (14.9)	0.933
Diastolic Blood Pressure (mmHg)	M	78.6 (8.9)	77.3 (7.9)	80.2 (8.9)	0.089	78.3 (10.2)	78.2 (8.4)	79.5 (8.6)	0.465
	W	74.5 (9.4)	73.4 (7.9)	75.8 (8.5)	0.278	73.2 (8.4)	74.5 (9.2)	75.2 (8.2)	0.389
Total cholesterol (mmol/L)	M	5.2 (1.0)	5.2 (1.0)	5.3 (0.9)	0.654	5.3 (1.1)	5.2 (1.0)	5.3 (0.9)	0.721
	W	5.4 (0.9)	5.4 (1.1)	5.4 (1.1)	0.996	5.5 (0.9)	5.4 (1.0)	5.5 (1.0)	0.778
HDL-cholesterol (mmol/L)	M	1.3 (0.3)	1.3 (0.3)	1.3 (0.4)	0.808	1.3 (0.4)	1.3 (0.3)	1.3 (0.3)	0.908
	W	1.7 (0.4)	1.6 (0.3)	1.6 (0.3)	0.235	1.7 (0.4)	1.7 (0.4)	1.6 (0.4)	0.444
LDL-cholesterol (mmol/L)	M	3.3 (0.9)	3.2 (0.9)	3.3 (0.9)	0.568	3.4 (0.9)	3.3 (0.9)	3.3 (0.9)	0.662
	W	3.2 (0.8)	3.3 (1.0)	3.3 (0.9)	0.786	3.2 (0.9)	3.2 (0.9)	3.4 (1.0)	0.728
Fasting Triglycerides (mmol/L)	M	1.3 (0.6)	1.3 (0.8)	1.5 (0.9)	0.224	1.3 (0.6)	1.3 (0.7)	1.5 (0.8)	0.066
	W	1.1 (0.6)	1.1 (0.5)	1.1 (0.4)	0.676	1.1 (0.5)	1.1 (0.6)	1.1 (0.5)	0.769
Fasting blood glucose (mmol/L)	M	5.6 (1.3)	5.4 (0.9)	5.5 (0.9)	0.446	5.6 (1.0)	5.4 (1.1)	5.6 (1.1)	0.439
	W	5.3 (0.9)	5.1 (0.6)	5.3 (0.9)	0.275	5.2 (0.8)	5.2 (0.8)	5.3 (1.0)	0.627

Data are presented as mean and standard deviation. Differences between SSBs consumers were assessed with ANOVA.

**Table 4:** Behavioral and social factors related to the different SSBs consumption patterns for men.

By Calorie Amount						By Frequency			
		None	Low	Moderate–High	*p*-Value	Never	Occasional	Daily	*p*-Value
Total		47.1 (42.1–52.3)	29.5 (24.9–34.3)	23.4 (19.0–28.0)		11.8 (8.3–15.3)	59.8 (55.0–64.9)	28.3 (23.8–33.1)	
Physical activity	Active	47.5 (40.2–54.9)	28.1 (21.5–35.1)	24.4 (18.2–31.3)		13.8 (9.0–19.6)	55.0 (47.4–62.2)	31.3 (24.2–38.3)	
	Minimally	46.5 (37.7–55.5)	33.9 (25.6–43.1)	19.7 (12.5–26.4)	0.532	9.4 (4.5–15.0)	66.9 (58.5–74.8)	23.6 (16.8–31.1)	0.352
	Inactive	47.5 (34.7–60.6)	23.7 (13.1–35.3)	28.8 (17.6–40.7)		11.9 (3.6–20.0)	57.6 (45.0–70.2)	30.5 (19.4–43.1)	
Smoking	Never	43.1 (28.6–57.1)	35.3 (23.3–49.0)	21.6 (10.5–34.2)		11.8 (3.6–21.3)	51.0 (36.2–64.9)	37.3 (24.5–52.1)	
	Past	50.9 (40.9–60.0)	27.7 (19.8–35.9)	21.4 (14.0–30.5)	0.775	15.2 (8.7–21.6)	59.8 (50.0–69.4)	25.0 (17.7–33.3)	0.353
	Current	45.9 (38.8–53.6)	29.0 (22.5–35.6)	25.1 (19.0–31.1)		9.8 (5.5–14.6)	62.3 (54.6–69.0)	27.9 (21.5–34.5)	
Alcohol intake	0	45.6 (38.6–52.4)	28.6 (22.0–35.0)	25.7 (19.6–32.0)		13.1 (8.6–18.1)	58.7 (52.0–65.9)	28.2 (21.9–34.4)	
	>0–20	48.2 (34.4–61.5)	23.2 (11.8–35.6)	28.6 (16.7–40.3)	0.181	12.5 (4.3–22.0)	62.5 (49.0–75.5)	25.0 (13.7–36.4)	0.788
	>20	50.0 (39.2–61.3)	35.7 (25.3–46.0)	14.3 (7.2–21.6)		8.3 (2.8–14.9)	60.7 (50.0–70.9)	31.0 (21.4–41.5)	
Total energy intake	<2000	52.0 (43.8–60.0)	27.7 (20.0–34.8)	20.3 (13.9–26.8)		15.5 (10.2–21.9)	64.2 (56.2–71.9)	20.3 (14.0–26.7)	
	2000–2500	46.4 (36.1–56.1)	27.8 (19.3–37.5)	25.8 (16.9–34.9)	0.460	11.3 (5.4–18.3)	59.8 (50.0–70.0)	28.9 (19.8–38.0)	0.012
	>2500	40.6 (30.6–50.0)	33.7 (23.9–42.9)	25.7 (17.4–34.1)		6.9 (2.1–12.4)	53.5 (43.6–63.9)	39.6 (29.9–49.1)	
Household income	High	46.5 (38.1–55.2)	29.5 (21.1–37.1)	24.0 (16.5–31.5)		11.6 (6.5–17.4)	67.4 (59.7–75.0)	20.9 (13.9–28.3)	
	Middle	50.9 (43.2–59.0)	29.9 (22.8–37.2)	19.2 (13.6–25.2)	0.160	10.2 (5.7–15.5)	59.3 (51.9–66.7)	30.5 (23.7–37.1)	0.026
	Low	36.0 (22.6–50.0)	28.0 (15.4–41.1)	36.0 (23.9–50.0)		18.0 (7.4–28.6)	42.0 (27.5–56.5)	40.0 (26.5–54.4)	
Education level	High	51.1 (43.1–59.0)	24.2 (17.8–30.9)	24.7 (18.2–31.2)		12.4 (7.9–17.7)	64.0 (56.4–71.3)	23.6 (17.6–29.7)	
	Medium	41.2 (30.9–51.5)	35.1 (25.6–45.5)	23.7 (15.6–31.8)	0.244	8.2 (3.3–14.1)	58.8 (49.5–69.1)	33.0 (23.3–42.9)	0.175
	Low	45.1 (33.9–57.1)	35.2 (24.3–47.3)	19.7 (10.1–29.0)		15.5 (7.7–24.4)	50.7 (39.2–62.3)	33.8 (22.5–44.9)	

Proportions are presented as percentages and a 95% Confidence Interval (95% CI). The chi-square test is used to determine different proportions.

**Table 5:** Behavioral and social factors related to the different SSBs consumption patterns for women.

		By Calorie Amount				By Frequency			
		None	Low	Moderate–High	*p*-Value	Never	Occasional	Daily	*p*-Value
Total		57.3 (52.6–62.8)	30.5 (25.4–35.0)	12.2 (9.0–15.7)		19.8 (15.7–23.9)	68.0 (63.2–72.5)	12.2 (8.9–15.6)	
Physical activity	Active	59.7 (51.3–67.3)	28.6 (21.9–35.5)	11.7 (6.7–17.2)		24.7 (18.0–32.1)	62.3 (54.5–70.0)	13.0 (7.8–18.5)	
	Minimally	56.5 (49.1–64.1)	29.2 (22.6–36.5)	14.3 (9.0–19.8)	0.476	17.3 (12.0–23.3)	73.8 (66.9–80.4)	8.9 (4.9–13.9)	0.061
	Inactive	53.2 (40.0–65.3)	38.7 (26.3–50.9)	8.1 (1.7–15.4)		14.5 (5.8–24.2)	66.1 (54.4–77.4)	19.4 (9.4–29.5)	
Smoking	Never	55.2 (41.0–68.6)	31.0 (19.6–44.4)	13.8 (5.3–23.1)		22.4 (11.9–34.0)	63.8 (50.0–75.9)	13.8 (5.4–23.9)	
	Past	58.4 (48.1–68.0)	32.7 (23.2–42.2)	8.9 (3.7–14.3)	0.813	19.8 (12.0–27.7)	64.4 (55.7–74.2)	15.8 (8.9–23.6)	0.597
	Current	57.3 (50.4–63.8)	29.3 (23.6–35.5)	13.3 (8.8–17.8)		19.1 (13.7–23.9)	70.7 (64.9–76.8)	10.2 (6.3–14.1)	
Alcohol intake	0	57.8 (51.7–63.7)	29.3 (23.7–34.9)	13.0 (9.2–17.0)		18.9 (14.2–23.5)	68.1 (62.7–73.3)	13.0 (8.9–17.2)	
	>0–20	55.4 (43.7–66.7)	35.1 (24.3–46.5)	9.5 (3.2–16.2)	0.861	17.6 (9.3–26.2)	77.0 (67.5–86.1)	5.4 (1.2–11.5)	0.039
	>20	57.5 (42.4–73.2)	30.0 (16.7–43.9)	12.5 (2.9–23.5)		30.0 (16.1–44.1)	50.0 (35.3–66.0)	20.0 (7.7–32.4)	
Total energy intake	<2000	61.9 (56.3–67.7)	25.9 (20.8–31.2)	12.2 (8.6–16.2)		22.3 (17.3–27.0)	67.6 (62.3–73.3)	10.1 (6.5–13.8)	
	2000–2500	46.8 (35.9–57.5)	40.5 (30.0–52.1)	12.7 (6.2–20.8)	0.025	12.7 (5.7–20.5)	72.2 (61.9–82.2)	15.2 (7.7–23.3)	0.049
	>2500	40.7 (22.2–60.0)	48.1 (28.0–66.7)	11.1 (0.0–24.2)		14.8 (3.4–28.1)	59.3 (40.6–78.6)	25.9 (10.0–44.8)	
Household income	High	59.4 (47.1–71.8)	31.9 (20.3–42.6)	8.7 (2.8–16.0)		14.5 (7.2–24.2)	76.8 (65.8–86.1)	8.7 (2.9–16.0)	
	Middle	60.3 (53.3–67.6)	29.1 (22.6–35.8)	10.6 (6.4–15.4)	0.348	19.6 (13.8–25.0)	67.7 (61.3–74.4)	12.7 (8.2–17.4)	0.446
	Low	51.6 (41.8–60.6)	31.7 (24.0–40.5)	16.7 (10.3–23.8)		23.0 (15.9–30.5)	63.5 (54.7–72.0)	13.5 (7.7–19.8)	
Education level	High	65.1 (58.2–72.3)	28.9 (21.4–36.1)	6.0 (2.4–9.8)		20.1 (13.9–26.5)	73.8 (66.9–80.8)	6.0 (2.6–9.9)	
	Medium	50.0 (42.4–57.8)	33.6 (26.6–40.5)	16.4 (11.2–22.8)	0.022	19.1 (13.3–25.4)	64.5 (56.6–72.0)	16.4 (10.8–22.6)	0.062
	Low	56.6 (45.7–67.4)	27.7 (18.2–37.5)	15.7 (8.3–23.9)		20.5 (12.1–29.1)	63.9 (53.6–73.5)	15.7 (7.8–23.9)	

Proportions are presented as percentages and a 95% Confidence Interval (95% CI). The chi-square test is used to determine different proportions.

**Table 6:** Behavioral and social factors related to the different SSBs consumption patterns, adjusting each variable by age and sex using a multinomial regression analysis.

		By Calorie Amount		By Frequency	
		Low	Moderate–High	Occasional	Daily
Physical activity	Active	1	1	1	1
	Minimally active	1.14 (0.79–1.64)	1.00 (0.64–1.57)	1.77 (1.12–2.78)	1.06 (0.60–1.86)
	Inactive	1.17 (0.72–1.90)	1.10 (0.61–1.96)	1.60 (0.86–2.98)	1.72 (0.84–3.53)
Smoking	Never	1	1	1	1
	Past	0.81 (0.48–1.37)	0.76 (0.39–1.48)	1.08 (0.57–2.05)	0.78 (0.37–1.67)
	Current	0.84 (0.52–1.36)	1.00 (0.55–1.82)	1.25 (0.69–2.27)	0.80 (0.40–1.62)
Alcohol intake	None	1	1	1	1
	Middle	0.91 (0.51–1.60)	1.67 (0.80–3.48)	1.55 (0.75–3.19)	0.74 (0.31–1.79)
	High	0.88 (0.56–1.38)	1.71 (0.93–3.12)	1.24 (0.69–2.22)	0.95 (0.49–1.85)
Total energy intake	<2000	1	1	1	1
	2000–2500	1.61 (1.07–2.43)	1.41 (0.86–2.31)	1.46 (0.85–2.49)	2.22 (1.17–4.21)
	>2500	2.08 (1.28–3.38)	1.55 (0.87–2.74)	1.45 (0.71–2.97)	3.80 (1.75–8.26)
Household income	High	1	1	1	1
	Middle	0.91 (0.60–1.38)	0.39 (0.56–1.54)	0.99 (0.57–1.73)	1.75 (0.89–3.41)
	Low	1.15 (0.68–1.95)	2.30 (1.24–4.26)	0.79 (0.41–1.50)	1.96 (0.89–4.33)
Education level	High	1	1	1	1
	Middle	1.66 (1.13–2.44)	1.75 (1.09–2.79)	1.14 (0.71–1.89)	2.25 (1.24–4.08)
	Low	1.35 (0.86–2.11)	1.38 (0.80–2.39)	0.88 (0.51–1.50)	1.78 (0.93–3.41)

Logistic regression analysis adjusting each variable by age and sex. Data are presented as Odds Ratio (OR) and 95% Confidence Interval (95% CI).

**Table 7:** Behavioral and social factors related to the different SSBs consumption patterns, fully adjusted using a multinomial regression analysis.

		By Calorie Amount		By Frequency	
		Low	Moderate–High	Occasional	Daily
Physical activity	Active	1	1	1	1
	Minimally active	1.24 (0.85–1.82)	1.04 (0.66–1.66)	1.78 (1.12–2.82)	1.20 (0.67–2.15)
	Inactive	1.26 (0.77–2.05)	1.10 (0.60–2.00)	1.69 (0.9–3.19)	2.02 (0.96–4.23)
Smoking	Never	1	1	1	1
	Past	0.83 (0.49–1.42)	0.74 (0.37–1.46)	1.15 (0.60–2.21)	0.86 (0.39–1.87)
	Current	0.90 (0.55–1.49)	1.01 (0.54–1.89)	1.30 (0.71–2.40)	0.92 (0.44–1.93)
Alcohol intake	None	1	1	1	1
	Middle	1.02 (0.57–1.84)	1.91 (0.89–4.10)	1.57 (0.74–3.32)	0.94 (0.37–2.37)
	High	1.02 (0.63–1.65)	2.00 (1.06–3.76)	1.28 (0.70–2.36)	1.18 (0.58–2.39)
Total energy intake	<2000	1	1	1	1
	2000–2500	1.71 (1.12–2.60)	1.55 (0.93–2.59)	1.15 (0.88–2.64)	2.44 (1.26–4.70)
	>2500	2.32 (1.39–3.85)	1.88 (1.03–3.44)	1.65 (0.78–3.45)	4.76 (2.11–10.76)
Household income	High	1	1	1	1
	Middle	0.80 (0.51–1.24)	0.83 (0.48–1.42)	1.00 (0.57–1.78)	1.52 (0.74–3.09)
	Low	0.94 (0.53–1.66)	2.05 (1.05–4.00)	0.77 (0.39–1.53)	1.54 (0.65–3.64)
Education level	High	1	1	1	1
	Middle	1.87 (1.24–2.84)	1.72 (1.03–2.86)	1.35 (0.80–2.26)	2.42 (1.27–4.62)
	Low	1.52 (0.94–2.48)	1.31 (0.71–2.41)	1.05 (0.59–1.87)	1.87 (0.91–3.85)

Logistic regression analysis adjusting each variable by age, sex, physical activity, smoking, alcohol intake, total energy intake, household income, and education level. Data are presented as Odds Ratio (OR) and 95% Confidence Interval (95% CI).

## Data Availability

The data presented in this study are available upon request from the corresponding author. The data are not publicly available.
